# Supplementary material for: Cryo-electron tomography of NLRP3-activated ASC complexes reveals organelle co-localization
Source: Nat Commun. 2023 Nov 9;14:7246. doi: 10.1038/s41467-023-43180-8 (PMC10636019; doi:10.1038/s41467-023-43180-8)
Supplement: Supplementary file 1 — Supplementary Information [file 41467_2023_43180_MOESM1_ESM.pdf]

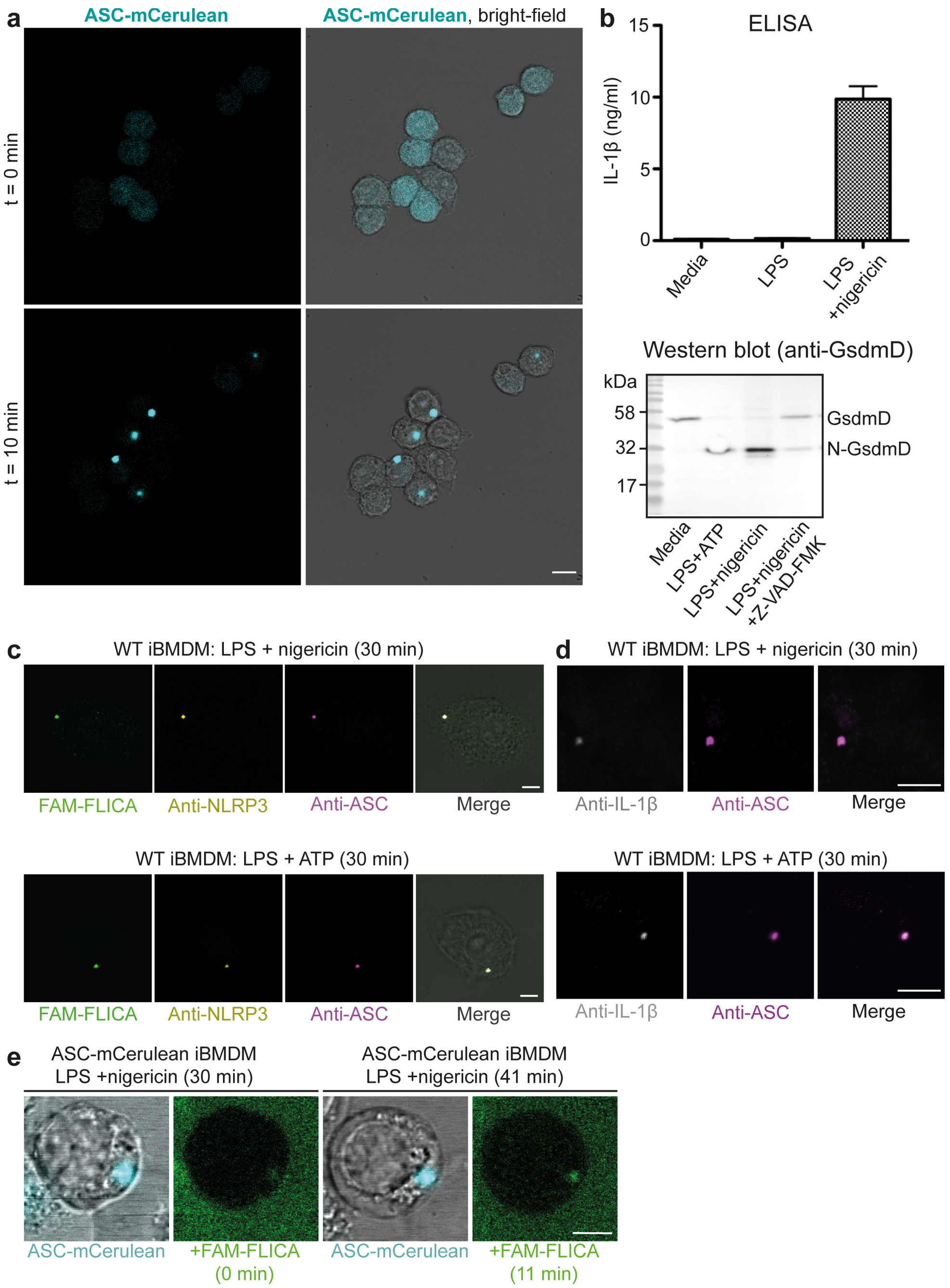

Supplementary Figure 1. Tracking NLRP3 inflammasome activation in iBMDMs with fluorescence confocal microscopy and biochemical assays. (a) Fluorescence and bright-field micrographs of live iBMDMs expressing ASC-mCerulean. ASC specks formed within 10 min of NLRP3 activation with LPS and nigericin. Scale bar, 10 μm. Images representative of three independent experiments. **(b)** Top, ELISA measuring IL-1β secretion in NLRP3-activated ASC-mCerulean iBMDMs; Bottom, Western blot showing proteolytic cleavage of GsdmD to yield the pore-forming N-terminal fragment (N-GsdmD). Error bars represent standard deviation from the mean (n = 3 independent experiments). **(c)** Fluorescence micrographs of WT iBMDMs stimulated with nigericin or ATP. Caspase-1 was detected with FAM-FLICA. NLRP3 and ASC were detected by immunostaining. Scale bars, 5 μm. Images representative of two independent experiments. **(d)** Immunofluorescence micrographs of WT iBMDMs stimulated with nigericin or ATP, stained for IL-1β and ASC. Scale bars, 5 μm. **(e)** Snapshots of live nigericin-stimulated ASC-mCerulean iBMDMs following addition of FAM-FLICA. Scale bar, 5 μm. Images representative of three independent experiments.

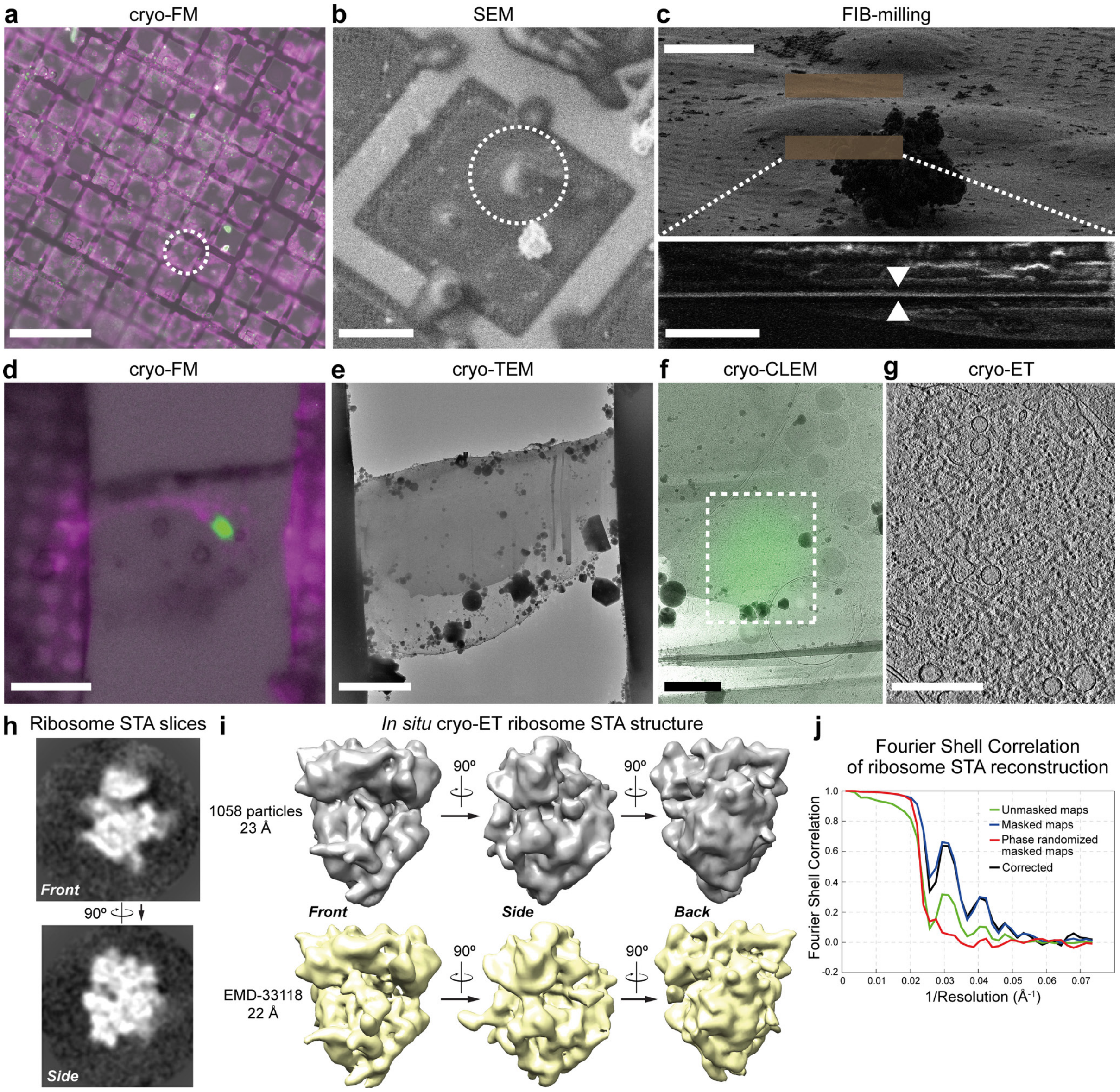

Supplementary Figure 2. Workflow for correlative cryo-light and electron microscopy (cryo-CLEM) **(a)** Cryo-fluorescence microscopy (cryo-FM) of ASC-mCerulean iBMDMs, with brightfield image overlaid. Cells were cultured on finder grids, primed with LPS, stimulated with nigericin for 30 min, labeled with MitoView and FAM-FLICA, vitrified, and imaged. The white circle denotes an area of interest. Scale bar: 300 µm. **(b)** Grids were transferred to a scanning electron microscope (SEM) fitted with a focused-ion-beam (FIB). The cell containing the area of interest, identified using the finder-grid markers, was imaged by SEM. Scale bar, 40 µm. **(c)** FIB-induced secondary electron images of the area shown in *(b)* before and after FIB milling: Upper panel, before FIB milling with initial target milling windows shown in brown (scale bar, 10 µm); lower panel, side-on view of the lamella after FIB milling (scale bar, 4 µm). **(d)** Cryo-FM image and overlaid brightfield image of a lamella containing an ASC speck. Scale bar, 10 µm. **(e)** Transmission cryo-EM map of the lamella shown in *(d)*. Scale bar, 7 µm. **(f)** Cryo-EM map overlaid with the cryo-FM map after registration and transformation. The region of interest for tilt-series acquisition is boxed. Scale bar, 1 µm. **(g)** Reconstructed tomographic slice acquired in the area boxed in *(f)*. Filaments and vesicles are visible. Scale bar, 300 nm. See Supplementary Movie 2 for a full tomogram. **(h)** Slices through a 23 Å resolution cryo-ET subtomogram averaging (STA) reconstruction of 1058 ribosomes from six tomograms of ASC-mCerulean iBMDMs. **(i)** Upper panels, volume representations of the STA reconstruction shown in *(h)*. Lower panels, *in situ* STA volumes of ribosomes from Ref. (1) with a 22 Å low-pass filter applied. **(j)** Fourier shell correlation plots for the ribosome STA reconstruction.

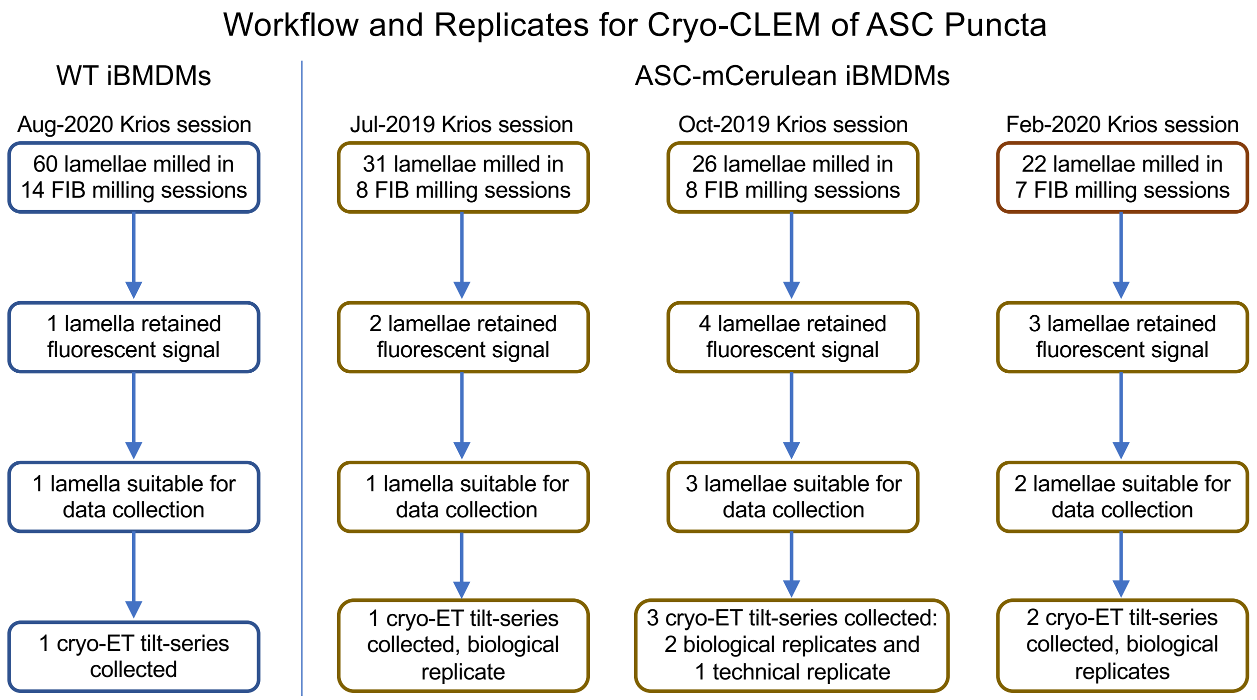

Supplementary Figure 3. Cryo-CLEM workflow statistics and replicates for imaging of ASC puncta

The cryo-CLEM workflow shown here was used to obtain the cryo-ET reconstructions of ASC puncta shown in **Figs. 1** and **2**.

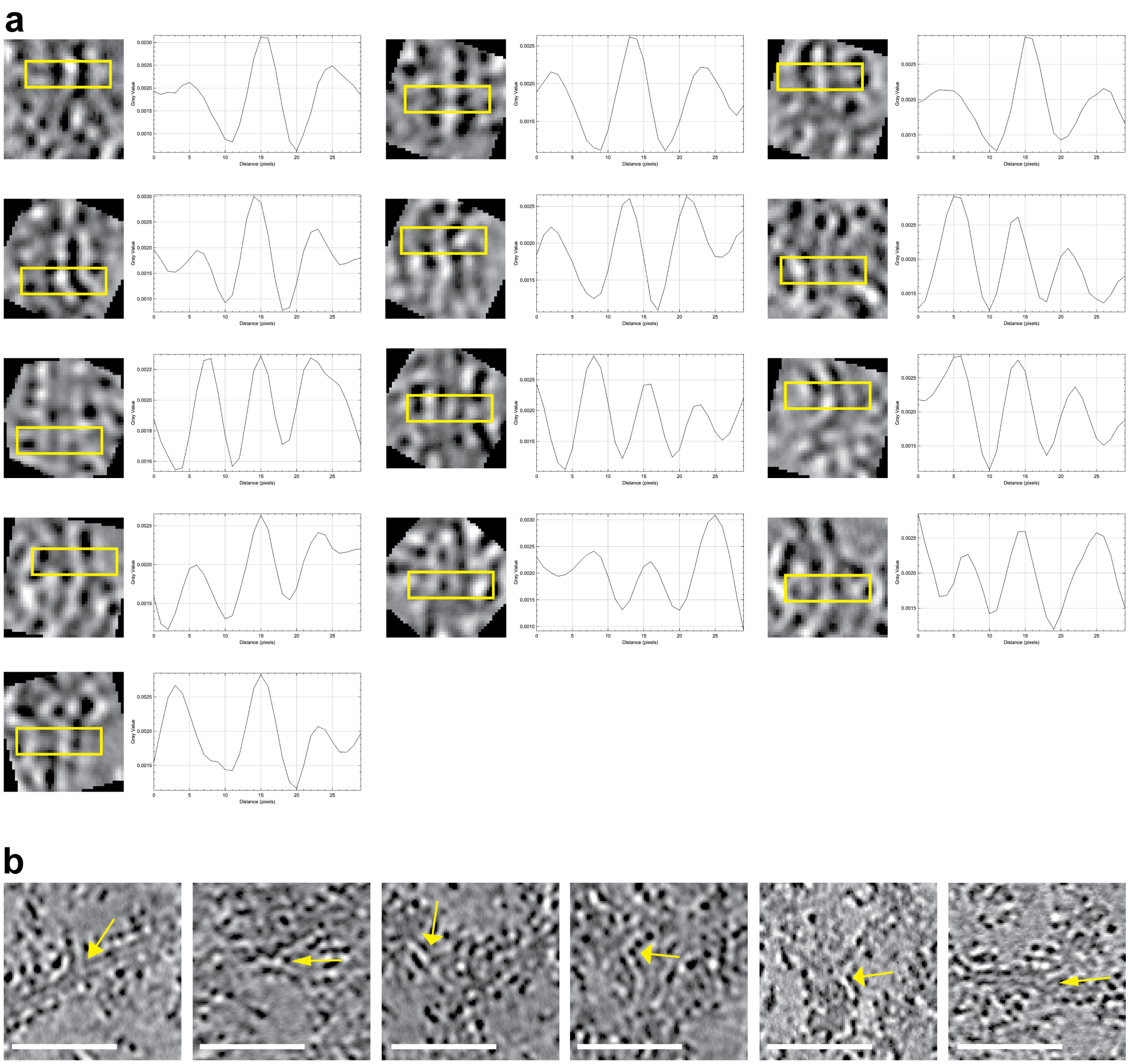

Supplementary Figure 4. Measurement sites for ASC filament properties on cryo-ET tomograms. (a) The thirteen 8-Å thick tomogram slices and associated density profiles plots that were used to measure the ASC filament core diameter (see **Fig. 2b,c**). The tomogram slices were restored with cryoCARE[2]. 30-by-8-pixel (24 x 6.4 nm) cross-section areas, boxed in yellow, were drawn in Fiji[3] perpendicular to the ASC filaments. Density profiles were plotted from these areas in Fiji with the Plot Profile function. The pixel size is 8 Å. **(b)** Tomogram slices showing filament branching angle measurement sites, marked by yellow arrows. Scale bars, 50 nm.

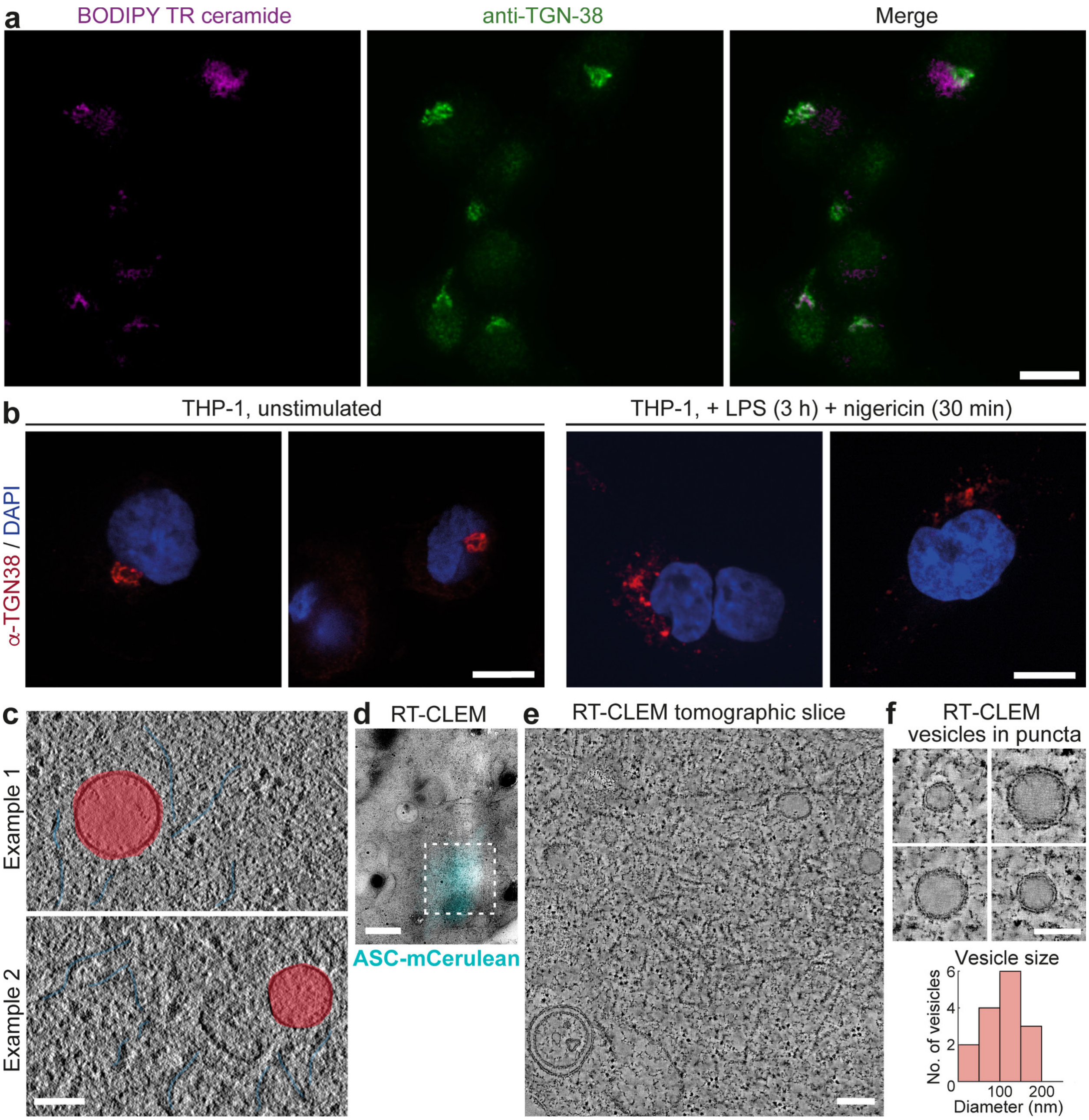

Supplementary Figure 5. Imaging of vesicles containing Golgi markers in ASC puncta. **(a)** Unstimulated WT iBMDMs stained with BODIPY TR and α-TGN38 antibody. Scale bar, 10 µm. Images are representative of two biological replicates. **(b)** THP-1 cells stained with anti-TGN38 antibody. Scale bars, 10 µm. Images are representative of two biological replicates. **(c)** Tomographic slices from an ASC-mCerulean speck showing vesicles adjacent to ASC filaments. Scale bar, 100 nm. **(d-f)** ASC-mCerulean iBMDMs stimulated with nigericin for 30 min, high-pressure frozen, chemically fixed, sectioned and imaged by CLEM at room temperature. (**d**) Low magnification EM map overlaid with ASC-mCerulean fluorescence. Scale bar, 1 µm. (**e**) 5.5 nm-thick tomographic slice of the region boxed in *(d)*. Scale bar, 200 nm. (**f**) Tomographic slices showing vesicles with low-density lumens within ASC-mCerulean puncta. Scale bar, 100 nm. The histogram shows the vesicle diameter distribution. Images in *(d-f)* are representative of three biological replicates.

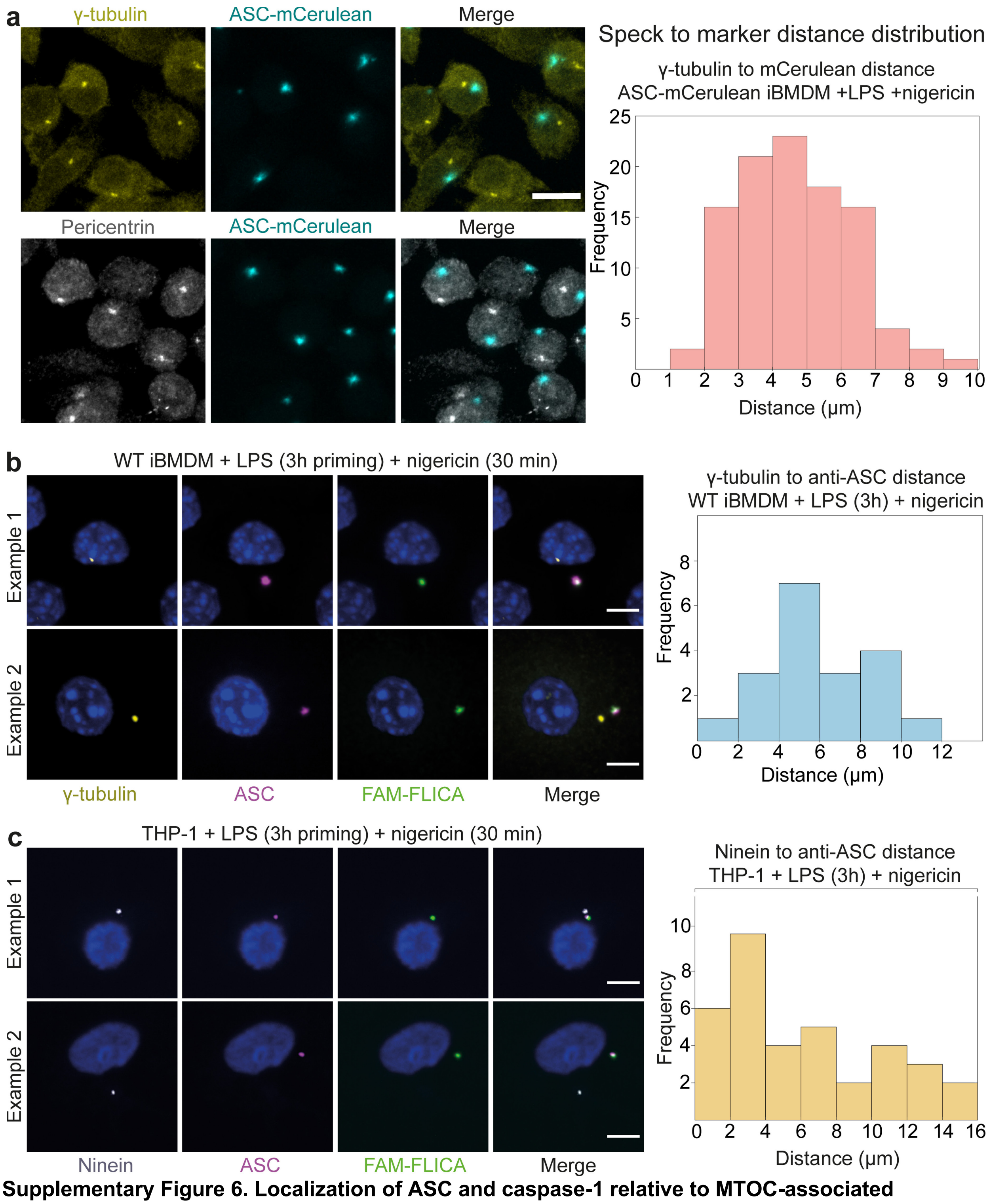

Supplementary Figure 6. Localization of ASC and caspase-1 relative to MTOC-associated components. (a) Fluorescence confocal micrographs of ASC-mCerulean iBMDMs stained with antibodies against γ-tubulin or pericentrin. Right, distance distribution between ASC-mCerulean and γ-tubulin foci. **(b)** Fluorescence micrographs of WT iBMDMs stained with FAM-FLICA and antibodies against γ-tubulin and ASC. Right, distance distribution between ASC/FAM-FLICA and anti-γ-tubulin fluorescence foci. **(c)** Fluorescence micrographs of THP-1 cells stained with FAM-FLICA and antibodies against ninein and ASC. Right, distance distribution between ASC/FAM-FLICA and anti-ninein foci. Scale bars, 10 μm. Images are representative of three independent experiments (>20 cells per replicate).

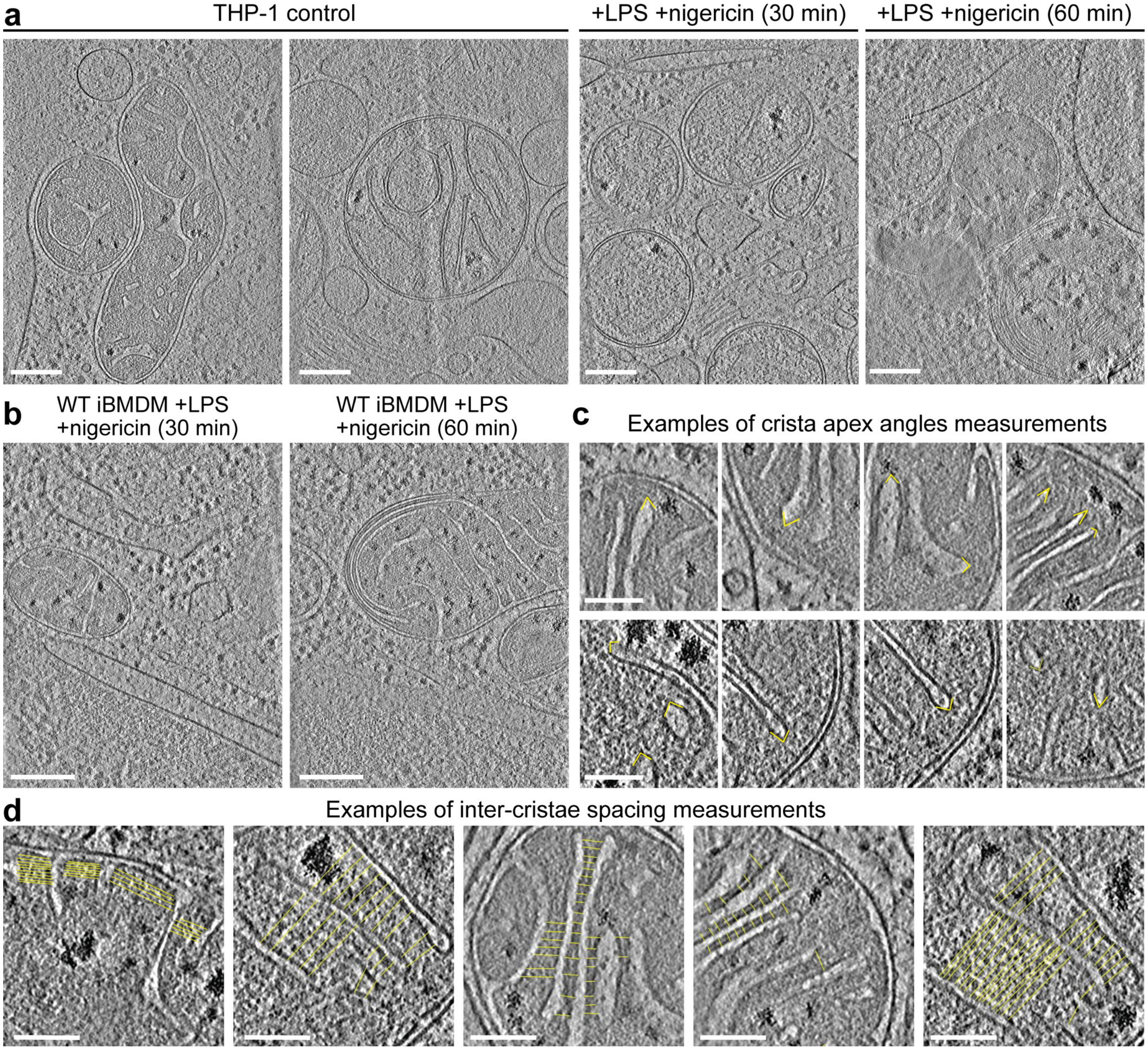

Supplementary Figure 7. Mitochondrial morphology of NLRP3-activated THP-1 cells and quantification of mitochondrial morphology. (a-b) Reconstructed cryo-ET tomographic slices of THP-1 cells, (**a**), and iBMDMs, (**b**), expressing WT ASC at different timepoints after stimulation with nigericin, or without stimulation (control). Scale bars, 200 nm. Images in *(a)* are from a single independent experiment. Images in *(b)* are from two independent experiments. **(c-d)** Cryo-ET tomographic slices showing how the mitochondrial cristae apex angles, (**c**), and inter-cristae spacings, (**d**), were measured for the quantitative analysis in **Fig. 4b**. Scale bars, 100 nm. Images are from three independent experiments.

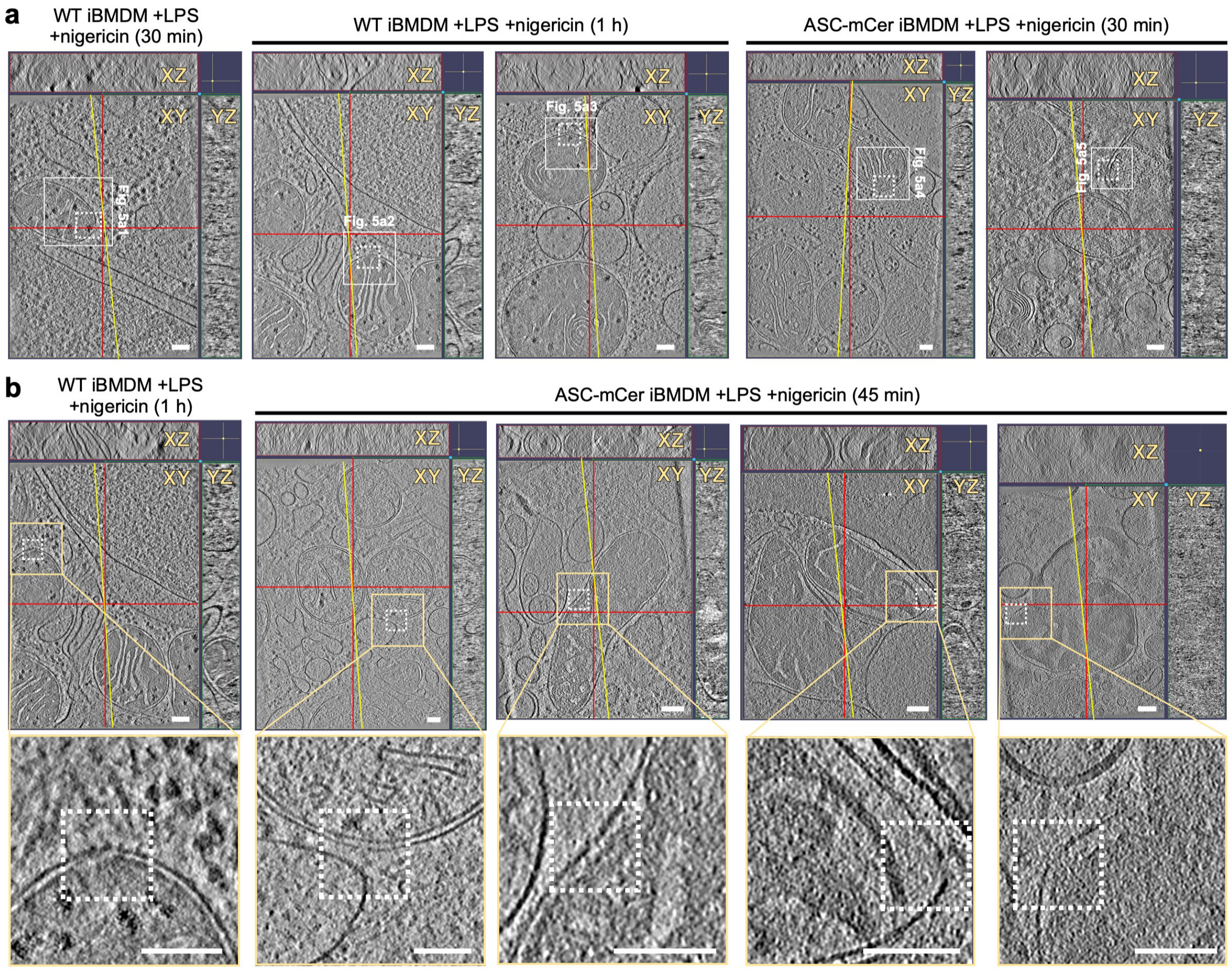

Supplementary Figure 8. Examples of discontinuities in the outer mitochondrial membrane (OMM). (a) Position of the tilt axis (yellow line) in the cryo-ET tomograms of nigericin-stimulated cells shown in **Fig. 5a,b**, shown here uncropped. Dashed boxes denote discontinuities in the OMM. Scale bars, 100 nm. **(b)** Additional examples of OMM gaps (dashed boxes). The tomogram slice on the left is from the same tomogram as the second tomogram slice from the left in (a). The third, fourth and fifth tomogram slices from the left were collected from the mitochondrial fraction following subcellular fractionation (see Methods). The position of the tilt axis is shown in yellow. Red lines indicate the x and y coordinates of the XZ and YZ sections, respectively. Closeups of each gap are shown below. All scale bars, 100 nm.

a

iBMDM type	Nigericin stimulation (min)	Gap diameter (nm)	Gap depth in z (nm)
ASC-mCerulean	30	10.5	10.9
ASC-mCerulean	30	14.6	12.2
ASC-mCerulean	30	18.2	13.6
ASC-mCerulean	30	10.6	13.6
WT	60	20.4	*
WT	60	17.4	17.1

*gap near the end of Z stack

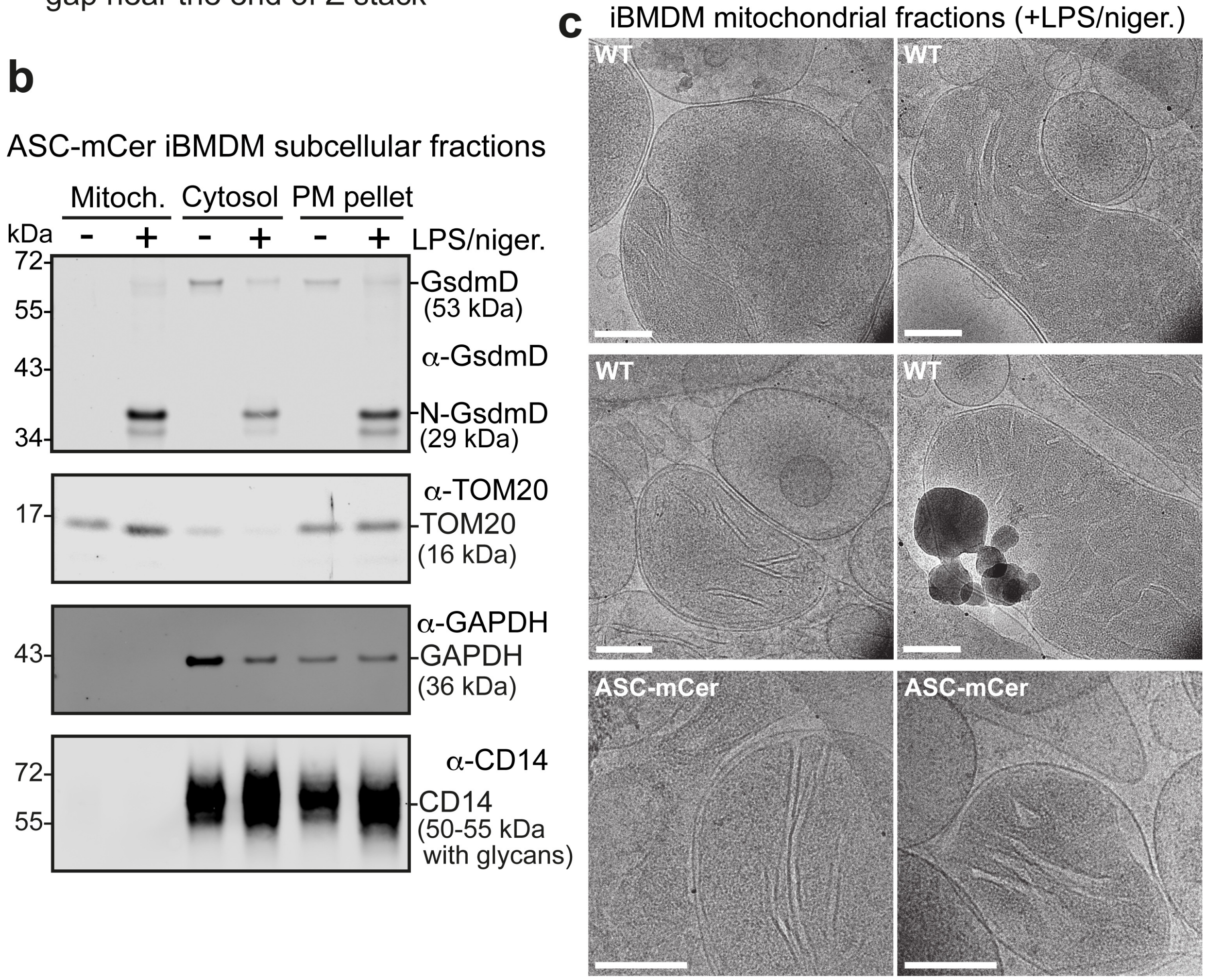

Supplementary Figure 9. Mitochondrial outer-membrane pore sizes and purification of the mitochondrial subcellular fraction. (a) Dimension of outer mitochondrial membrane pore in cryo-ET reconstructions of iBMDMs stimulated with LPG and nigericin. Two of the pores from ASC-mCerulean iBMDMs and WT iBMDMs are shown in **Fig. 5a,b**. **(b)** Immunoblots of subcellular fractions of ASC-mCerulean iBMDMs 60 min after stimulation with LPS and nigericin. The GsdmD N-terminal domain (N-GsdmD) is enriched in the mitochondrial fraction. Shown below are immunoblots for Tom20 (a mitochondrial protein), GAPDH (a cytosolic protein), and CD14 (a plasma membrane protein). **(c)** Cryo-EM images of the purified mitochondrial fraction. Scale bars, 200 nm. Images in *(a-c)* are representative of three independent experiments.

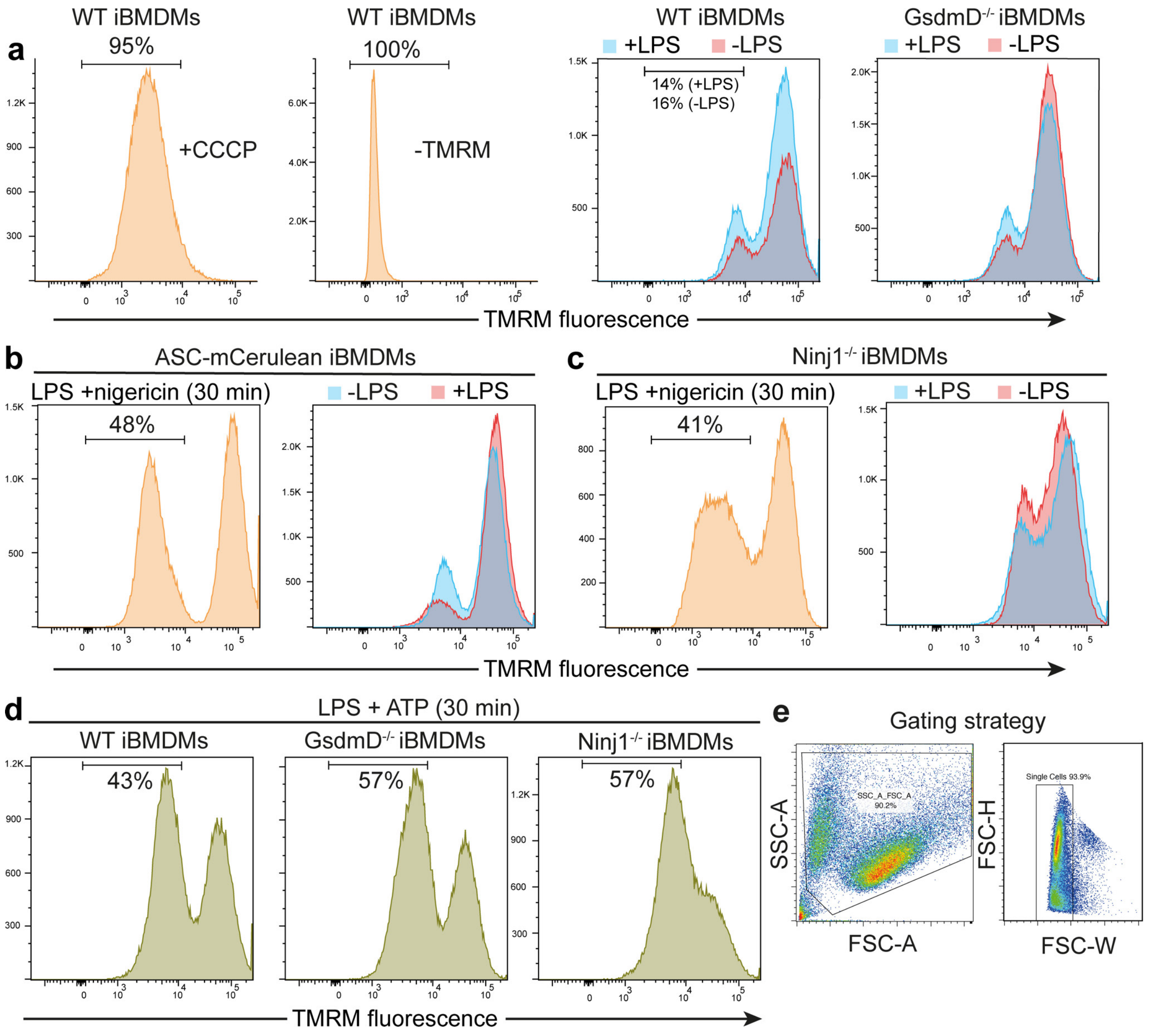

Supplementary Figure 10. GsdmD contributes to mitochondrial membrane potential loss following NLRP3 activation. (a) Flow cytometry controls supporting **Fig. 5c,d**. From left to right: cells treated with carbonyl cyanide 3-chlorophenylhydrazone (CCCP), a mitochondrial uncoupler, and stained with TMRM (positive control); cells without TMRM staining (negative control); WT iBMDMs stained with TMRM with or without LPS priming (also shown in Fig. 5C); GsdmD$^{-/-}$ iBMDMs stained with TMRM with or without LPS priming. Vertical axes indicate cell count. **(b)** 48% of ASC-mCerulean iBMDMs had loss of mitochondrial membrane potential following LPS priming and 30 min nigericin stimulation. Background controls without nigericin stimulation, with or without LPS priming are shown. **(c)** 41% of Ninj1$^{-/-}$ iBMDMs had loss of mitochondrial membrane potential following LPS priming and 30 min nigericin stimulation. Controls without nigericin stimulation, with or without LPS priming are shown. **(d)** Loss of mitochondrial membrane potential following priming with LPS and stimulation with ATP. **(e)** Gating strategy used to identify cells with loss of mitochondrial membrane potential. Side scatter area (SSC-A) and forward scatter area (FSC-A) were used to exclude debris. Forward scatter height (FSC-H) and forward scatter width (FSC-W) were used to exclude multiplet cells. Data shown in *(a-e)* are representative of three independent experiments.

Supplementary References

1. Eisenstein, F. et al. Parallel cryo electron tomography on in situ lamellae. *Nat Methods* **20**, 131-138 (2023).
2. Buchholz, T.O. et al. Content-aware image restoration for electron microscopy. *Methods Cell Biol* **152**, 277-289 (2019).
3. Schindelin, J. et al. Fiji: an open-source platform for biological-image analysis. *Nat Methods* **9**, 676-82 (2012).
