# Supplementary material for: Cryo-electron tomography of NLRP3-activated ASC complexes reveals organelle co-localization
Source: Nat Commun. 2023 Nov 9;14:7246. doi: 10.1038/s41467-023-43180-8 (PMC10636019; doi:10.1038/s41467-023-43180-8)
Supplement: Supplementary file 3 — Description of additional supplementary files [file 41467_2023_43180_MOESM3_ESM.pdf]

Description of additional supplementary files

Supplementary Movie 1 : Live-cell fluorescence imaging of iBMDMs stimulated with nigericin in the absence or presence of caspase-1 inhibitor Z-VAD-FMK (left and right, respectively).

Supplementary Movie 2 : Z-stack series of reconstructed cryo-ET volume of the ASCmCerulean punctum shown in Figs. 1 and 2 overlaid with the 3-D segmented model shown in Fig. 2.

Supplementary Movie 3 : Distribution of BODIPY TR ceramide during ASC speck formation in live iBMDMs expressing ASC-mCerulean (left), or costained with FAMFLICA (right).

Supplementary Movie 4 : Immunofluorescence micrograph Z-stack series of LPS-primed WT iBMDMs without nigericin stimulation (left), and after 30 min nigericin stimulation (right). Images from these Z-stack series are shown in Fig. 3b. Anti-NLRP3 partially colocalizes with anti-TGN38, and anti-TGN38 fluorescence disperses in the nigericinstimulated cells. Pink, antiNLRP3. Yellow, anti-TGN38. Scale bars, 10 µm.
