# Supplementary material for: Cryo-electron tomography of NLRP3-activated ASC complexes reveals organelle co-localization
Source: Nat Commun. 2023 Nov 9;14:7246. doi: 10.1038/s41467-023-43180-8 (PMC10636019; doi:10.1038/s41467-023-43180-8)
Supplement: Supplementary file 8 — Reporting Summary [file 41467_2023_43180_MOESM8_ESM.pdf]

nature portfolio

Corresponding author(s): Clare E. Bryant
Yorgo Modis

Last updated by author(s): Oct 24, 2023

Reporting Summary

Nature Portfolio wishes to improve the reproducibility of the work that we publish. This form provides structure for consistency and transparency in reporting. For further information on Nature Portfolio policies, see our Editorial Policies and the Editorial Policy Checklist.

Statistics

For all statistical analyses, confirm that the following items are present in the figure legend, table legend, main text, or Methods section.

n/a	Confirmed	
☐	☒	The exact sample size (*n*) for each experimental group/condition, given as a discrete number and unit of measurement
☐	☒	A statement on whether measurements were taken from distinct samples or whether the same sample was measured repeatedly
☐	☒	The statistical test(s) used AND whether they are one- or two-sided<br>*Only common tests should be described solely by name; describe more complex techniques in the Methods section.*
☒	☐	A description of all covariates tested
☒	☐	A description of any assumptions or corrections, such as tests of normality and adjustment for multiple comparisons
☐	☒	A full description of the statistical parameters including central tendency (e.g. means) or other basic estimates (e.g. regression coefficient) AND variation (e.g. standard deviation) or associated estimates of uncertainty (e.g. confidence intervals)
☐	☒	For null hypothesis testing, the test statistic (e.g. *F*, *t*, *r*) with confidence intervals, effect sizes, degrees of freedom and *P* value noted<br>*Give P values as exact values whenever suitable.*
☒	☐	For Bayesian analysis, information on the choice of priors and Markov chain Monte Carlo settings
☒	☐	For hierarchical and complex designs, identification of the appropriate level for tests and full reporting of outcomes
☒	☐	Estimates of effect sizes (e.g. Cohen's *d*, Pearson's *r*), indicating how they were calculated

Our web collection on statistics for biologists contains articles on many of the points above.

Software and code

Policy information about availability of computer code

Data collection	CLEM data collection:<br>-ICY v1.9.5.1 (DOI:10.1038/nmeth.2075)<br>-eC-CLEM plugin (DOI:10.1038/nmeth.4170).<br>Cryo-ET data collection:<br>-SerialEM v3.8.0 (DOI:10.1016/j.jsb.2005.07.007)<br>-SubTOM suite (https://github.com/DustinMorado/subTOM)<br>FACS data collection: EC800 Analyzer v1.3.6 (SONY/iCyt).
Data analysis	cryoET subtomogram averaging: Warp, RELION v3.1, relion_star_downgrade.py (dynamo2m. GitHub, DOI:10.5281/zenodo.4064754 (2020)), dynamo2warp.py (dynamo2m. GitHub, DOI:10.5281/zenodo.4064754 (2020)), relionsubtomo2ChimeraX.py (Bui, K.H. & Wagner, T. subtomo2Chimera. DOI:10.5281/zenodo.6820119 (2022)).<br>cryoET analysis: UCSF Chimera v1.16, Dynamo, Fiji v2.1.0, IMOD (DOI:10.1006/jsbi.1996.0013), cryoCARE, TomoSegMemTV (DOI:10.1016/j.jsb.2014.02.015), MATLAB 2020 (MathWorks, Inc.), Prism v9.2.0 (GraphPad), Igor (WaveMetrics, Inc.).<br>Light microscopy analysis: Morph_ROI.ijm and FRAP_Measure sFRAP_Measure.ijm (Boulanger, J. Cryo-electron tomography of NLRP3-activated ASC complexes reveals organelle co-localization. GitHub, DOI:10.5281/zenodo.10033040 (2023)), Python 3.0 (www.python.org).<br>Flow cytometry data analysis: Flowjo10 (TreeStar, Ashland, Oregon, USA).

For manuscripts utilizing custom algorithms or software that are central to the research but not yet described in published literature, software must be made available to editors and reviewers. We strongly encourage code deposition in a community repository (e.g. GitHub). See the Nature Portfolio guidelines for submitting code & software for further information.

Data

Policy information about availability of data

All manuscripts must include a data availability statement. This statement should provide the following information, where applicable:

- Accession codes, unique identifiers, or web links for publicly available datasets
- A description of any restrictions on data availability
- For clinical datasets or third party data, please ensure that the statement adheres to our policy

Representative electron tomograms were deposited in the Electron Microscopy Data Bank (EMDB) with accession codes EMD-13585 and EMD-13586.

Research involving human participants, their data, or biological material

Policy information about studies with human participants or human data. See also policy information about sex, gender (identity/presentation), and sexual orientation and race, ethnicity and racism.

Reporting on sex and gender	N/A
Reporting on race, ethnicity, or other socially relevant groupings	N/A
Population characteristics	N/A
Recruitment	N/A
Ethics oversight	N/A

Note that full information on the approval of the study protocol must also be provided in the manuscript.

Field-specific reporting

Please select the one below that is the best fit for your research. If you are not sure, read the appropriate sections before making your selection.

☒ Life sciences ☐ Behavioural & social sciences ☐ Ecological, evolutionary & environmental sciences

For a reference copy of the document with all sections, see nature.com/documents/nr-reporting-summary-flat.pdf

Life sciences study design

All studies must disclose on these points even when the disclosure is negative.

Sample size	No sample-size calculations were performed because the samples sizes chosen out of practical consideration resulted in statistical differences that were large enough that sample sizes were unambiguously sufficient.
Data exclusions	No data was excluded from the analyses in this study.
Replication	All attempts at replication were successful and results from all replicates were self-consistent. Three independent experiments were performed for each experiment, except where noted otherwise in the figure legends and supplementary figure legends.
Randomization	There were no human or animal participants in this study. Random sample allocation did not apply because samples were not subjected to co- or multivariate analysis and the statistical tests used were chosen to detect and compensate for any non-random allocation.
Blinding	The investigators were not blinded to group allocation because the group was too small to be blinded.

Reporting for specific materials, systems and methods

We require information from authors about some types of materials, experimental systems and methods used in many studies. Here, indicate whether each material, system or method listed is relevant to your study. If you are not sure if a list item applies to your research, read the appropriate section before selecting a response.

Materials & experimental systems

n/a	Involved in the study
☐	☒ Antibodies
☐	☒ Eukaryotic cell lines
☒	☐ Palaeontology and archaeology
☒	☐ Animals and other organisms
☒	☐ Clinical data
☒	☐ Dual use research of concern
☒	☐ Plants

Methods

n/a	Involved in the study
☒	☐ ChIP-seq
☐	☒ Flow cytometry
☒	☐ MRI-based neuroimaging

Antibodies

Antibodies used

The following primary antibodies were used:
Rabbit monoclonal anti-mouse GsdmD [EPR19828], 1:1,000 dilution (Abcam, ab209845, RRID:AB_2783550)
Rabbit anti-ASC rabbit polyclonal pAb AL177, 1:200 dilution (AdipoGen, AG-25B-0006, RRID:AB_2490440)
Rabbit anti-ASC monoclonal ASC/TMS1(D2W8U), 1:800 dilution (Cell Signalling Technology, 67824, RRID:AB_2799736)
Goat anti-NLRP3 polyclonal, 1:200 dilution (Abcam, ab4207, RRID:AB_955792)
Goat anti-IL-1b polyclonal, 1:500 dilution (R&D Systems, AF-401-NA, RRID:AB_416684)
Mouse anti-γ-tubulin monoclonal, 1:400 dilution (Sigma-Aldrich, T6557, RRID:AB_477584)
Rabbit anti-TGN38 polyclonal, 1:250 dilution (Novus Biologicals, NBP1-03495, RRID:AB_1522533)
Rabbit anti-TOM20 FL-145 polyclonal, 1:500 dilution (Santa Cruz Biotechnology, sc-11415, RRID:AB_2207533)
Rabbit monoclonal anti-mouse CD14, 1:1,000 dilution (Abcam, ab221678, RRID:AB_2935854)
Mouse monoclonal anti-GAPDH, 1:5,000 dilution (Proteintech, 60004-1-Ig, RRID: AB_2107436)

The following secondary antibodies were used:
Goat anti rabbit IgG-HRP (1:1000, Santa Cruz Biotechnology, sc-2004, RRID:AB_631746)
Alexa Fluor 488-labelled donkey anti-goat IgG (H+L) (1:500, ThermoFisher, A11055, RRID:AB_2534102)
Alexa Fluor 555-labelled goat anti-rabbit IgG(H+L) (1:500, ThermoFisher, A21428 RRID:AB_141784)
Alexa Fluor 568-labelled goat anti-mouse IgG (H+L) (1:500, ThermoFisher, A11004, RRID:AB_2534072)
Alexa Fluor 647-labelled goat anti-rabbit IgG (H+L) (1:500, ThermoFisher, A21244, RRID:AB_2535812)

Validation

Abcam ab209845: validated by manufacturer by Western blotting with WT RAW 264.7 and Gsdmd KO RAW 264.7 cell samples as positive and negative controls, respectively [https://www.abcam.com/products/primary-antibodies/gsdmd-antibody-epr19828-ab209845.html]
AdipoGen AG-25B-0006: validated by manufacturer by Western blotting with samples from multiple cell lines [https://adipogen.com/ag-25b-0006-anti-asc-pab-al177.html]. Product application described in DOI: 10.1016/s1074-7613(04)00046-9.
Cell Signalling Technology 67824: validated by manufacturer by Western blotting, immunohistochemistry, immnoprecipitation, flow cytometry, and fluorescence microscopy [https://www.cellsignal.com/products/primary-antibodies/asc-tms1-d2w8u-rabbit-mab/67824].
Abcam ab4207: validated by manufacturer by Western blotting and flow cytometry using WT and NLRP3 KO THP-1 cells [https://www.abcam.com/products/primary-antibodies/nlrp3-antibody-ab4207.html].
R&D Systems AF-401-NA: validated by manufacturer by Western blotting in THP-1 and RAW 264.7 cells, fluorescence microscopy, histochemistry, and IL-1b neutralization assay [https://www.rndsystems.com/products/mouse-il-1beta-il-1f2-antibody_af-401-na].
Sigma-Aldrich T6557: validated by manufacturer by Western blotting, immunofluorescence microscopy in multiple mouse and human cell lines [https://www.sigmaaldrich.com/GB/en/product/sigma/t6557]. Application demonstrated by Western blotting in DOI:10.1371/journal.ppat.1000492 and DOI: 10.1371/journal.pone.0006651
Novus Biologicals NBP1-03495: validated by manufacturer by immunofluorescence microscopy in mouse embryonic fibroblasts and human melanoma C32 cells [https://www.novusbio.com/products/tgn38-antibody_nbp1-03495#reviews-publications]. Application shown in DOI:10.1016/j.cell.2021.11.011
Santa Cruz Biotechnology sc-11415: Application demonstrated in DOI:10.7150/thno.32637, DOI:10.1242/jcs.195339, and DOI:10.15252/embj.201593727
Abcam ab221678: validated by manufacturer by flow cytometry, Western blotting, immunofluorescence microscopy, and fluorescence spectrum analysis [https://www.abcam.com/products/primary-antibodies/cd14-antibody-epr21847-ab221678].
Proteintech 60004-1-Ig: validated by manufacturer by Western blotting, flow cytometry, and immunofluorescence microscopy [https://www.ptglab.com/products/GAPDH-Antibody-60004-1-Ig.htm].

Eukaryotic cell lines

Policy information about cell lines and Sex and Gender in Research

Cell line source(s)

THP-1 cells were originally generated from a male acute monocytic leukemia patient. The THP-1 cells used in this study were obtained from the European Collection of Authenticated Cell Cultures (ECACC).
ASC-mCerulean iBMDM cells were obtained from Dr. Eicke Latz and are described in Stutz et al. (2013), DOI:10.1007/978-1-62703-523-1_8. The genotype of the ASC0mCerulean cells is NLRP3 KO, mNLRP3-Flag-IRES GFP, 00389 CMV-hAsc (wt)-mCerulean and the donor animal (CL14) was female.

Authentication	None of the cell lines were authenticated.
Mycoplasma contamination	Cell lines were routinely tested for mycoplasma contamination using the MycoAlert detection kit (Lonza). All tests were negative.
Commonly misidentified lines (See ICLAC register)	No commonly misidentified cell lines were used.

Flow Cytometry

Plots

Confirm that:

- [x] The axis labels state the marker and fluorochrome used (e.g. CD4-FITC).
- [x] The axis scales are clearly visible. Include numbers along axes only for bottom left plot of group (a 'group' is an analysis of identical markers).
- [x] All plots are contour plots with outliers or pseudocolor plots.
- [x] A numerical value for number of cells or percentage (with statistics) is provided.

Methodology

Sample preparation	Live cultured THP-1 and iBMDM cells were counted without chemical fixation treatment.
Instrument	Eclipse flow cytometer (Sony Biotechnology) equipped with 3 lasers (5 colours), and a multi-tube and plate sampler.
Software	Data collection: EC800 Analyzer v1.3.6 (SONY/iCyt); Data analysis: Flowjo10 (TreeStar, Ashland, Oregon, USA).
Cell population abundance	Post-sort fractions were not collected or assessed for purity.
Gating strategy	Live cells were gated on based on FSC and SSC as shown in the attached figure exemplifying the gating strategy; no further gating was performed.

- [x] Tick this box to confirm that a figure exemplifying the gating strategy is provided in the Supplementary Information.
